# Improving Admission Clerking and Physical Health Checks in Acute Psychiatric Inpatients. A Quality Improvement Project (QIP)

**DOI:** 10.1192/bjo.2026.11255

**Published:** 2026-06-30

**Authors:** Rafia Mubashir, Bushra Arooj

**Affiliations:** Battersea Bridge House Hospital, London, United Kingdom

## Abstract

**Aims:**

It has been suggested that early admission clerking, PHE and MSE play an essentialrole in the assessment and management of psychiatric disorders in patients. Middle-aged patients can have pre-existing multiple physical comorbidities.

Taking detailed history, Admission bloods, ECG and physical health checks help us to identify any underlying physical illness which can impact the mental health and recovery of the patients.

Aims were to assess whether the standard admission protocol is completed within 72 hoursof admission in an acute psychiatric inpatient setting.

Expected Standards (5 criteria)

1. Admission clerking (history taking).

2. Mental State Examination (MSE).

3. Physical examination.

4. ECG.

5. Routine admission blood tests.

**Methods:**

Battersea Bridge House Hospital is a 22-patient acute mental health facility, which consists of three wards:

Blake Ward–Female patients only, capacity 6 patients.

Browning Ward–Female patients only, capacity 10 patients.

Hardy Ward–Male patients only, capacity 6 patients.

Set standards: Locally and NHS trust, all admission protocols, consisting of five criteria, need to be completed within 72 hours.

Current standards: The current standard on admission to mental health at present is a timely clerking, mental state assessment, physical examination, routine bloods and ECG.

Data collected retrospectively from electronic clinical records of inpatient admissions in three psychiatry wards.

Duration: From January to April, the inpatient admissions at Battersea Bridge House Hospital; there were 43 patients in total, admitted in this time duration.

Study: A retrospective review of electronic patient records, for all inpatients admitted within women and men secure services at BBH from 1 January to April 2025 (n=43).

Forty-three records were audited against the set standards using current standards that were based on the Hospital accepted guidelines for admission protocol.

**Results:**

Patients were categorised based on completion of all five criteria within 72 hours:

- Green (Timely admission protocol):27 patients (62.8%).
- Red (Delayed admission protocol):14 patients (33.6%).

Exceptions:

- 2 patients (4.7%) refused blood tests.
- 1 male patient admitted for only 3 days (temporary admission).
- 1 female patient persistently refused blood tests during admission.

Reasons for Delay: For 41 patients, delays were documented and attributed to:

- Patient agitation or acute mental disturbance at presentation.
- Initial refusal of blood tests or ECG.
- Delayed consent (later acceptance after initial refusal).
- For 2 patients, reasons for delay werenot documented.

**Conclusion:**

There were delays in admission blood as many patients, depending upon the severity of their mental health issues and status, refused for timely admission bloods. Some refused bloods due to phobias and beliefs. Somepatients never provided reasons of declining bloods. Delays were also observed in getting the results back from the laboratory and our senior team is working on it to resolve this matter soon.

Summary of Improvements: A sharp rise in timely protocol implementation (27.1% or 1.76-fold increase); a reduction in delays (1.39-fold decreased); a notable drop in patient refusal (4-fold decrease).

Good rapport and active physical health handover with PHN result in better compliance of patients.

Key Learning Points:

- Timely physical health screening on admission is achievable in most cases.
- Patient capacity, consent, and mental state significantly influence compliance.
- Clear documentation of reasons for delay is essential.
- Routine ECG availability on the ward supports timely assessment.

TakeHome Message: Our audits offer the opportunity to compare our performance with other UK mental health sites. It is useful to note that, while the Royal College of Psychiatristsrecommends that all psychiatric inpatients receive a full physical examination within 24 hours of admission, in practice this is very difficult to achieve in mental health settings.

For instance, in a snapshot audit at Prospect Park Hospital (Hassan et al., 2019), 70.3% of patients received a physical exam on admission. After introducing a more standardised physical health protocol, the percentage of compliance increased, although minimally, to 75%.

Another case in point on the challenge of completing consistently physical health exams in mental health settings, comes from a re-audit by Oxford Health NHS Foundation Trust (2013), where they report fluctuating levels of physical health examinations over a three-year period: 90% in 2011, 96% in 2012, which dropped to 82% in 2013.

- While the overall compliance for physical health checks at Battersea Bridge House increased from 62.4% to 96.6% after intervention.

It is essential that all patients must have full physical health screening on admissions and timely documentation. A prompt handover to Physical health nurse and junior doctors results in good compliance.

Any barriers to set criteria of the five admission protocols should be identified with regular audits and QIPs.

Some limitations will continue to exist despite good rapport and prompt intervention such as religious beliefs and phobias.

References

Hassan, S. et al. (2019) Improving physical healthcare provided to psychiatric inpatients at an acute mental health trust. BMJ open quality. [Online] 8 (3), e000537.

Oxford Health NHS Foundation Trust (2019) Supporting inpatient mental health physical health assessments: Addressing gaps in routine care [Audit report], National Institute for Health and Care Excellence, available at: https://www.nice.org.uk/media/default/sharedlearning/696_696supportinginfo.pdf (Accessed: June 26, 2025).

